# Correction to: cRel and Wnt5a/Frizzled 5 Receptor-Mediated Inflammatory Regulation Reveal Novel Neuroprotectin D1 Targets for Neuroprotection

**DOI:** 10.1007/s10571-022-01239-y

**Published:** 2022-06-27

**Authors:** Jorgelina M. Calandria, Khanh V. Do, Sayantani Kala-Bhattacharjee, Andre Obenaus, Ludmila Belayev, Nicolas G. Bazan

**Affiliations:** 1 Neuroscience Center of Excellence, School of Medicine, Louisiana State University Health New Orleans, New Orleans, LA 70112 USA; 2 Department of Pediatrics, University of California, Irvine, CA 92618 USA; 3 Present Address: Faculty of Medicine, PHENIKAA University and PHENIKAA Research and Technology Institute (PRATI), A&A Green Phoenix Group JSC, Hanoi, Vietnam

## Correction to: Cellular and Molecular Neurobiology 10.1007/s10571-022-01231-6

The original version of this article, unfortunately, contained errors in the figures (Figs. 1–6) and to the caption of Fig. 5i. The correct Figs. 1, 2, 3, 4, 5 and 6 are presented with this erratum, and the corrected Fig. 5i caption is given below:

**Fig. 1:**
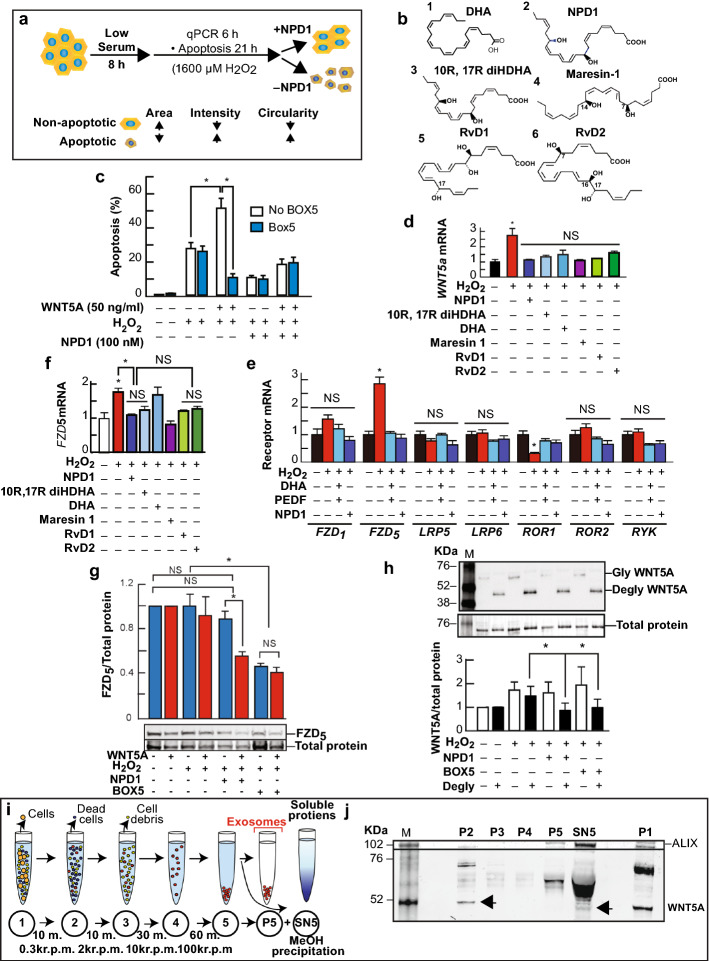
Docosanoids counteract UOS-dependent increase in Wnt5a, FZD_5_ expression, and apoptosis. (**a**) Design to determine apoptotic cells. (**b**) DHA (**1**) and its derivatives: NPD1 (**2**), 10R, 17R diHDHA (**3**), Maresin-1 (**4**), RvD1 (**5**), and RvD2 (**6**) counteracted these effects. (**c, e, and f**) Docosanoids prevented transcription increase in cells undergoing UOS. SYBR green real-time PCR was used to determine semi-quantitatively Wnt5a (**c**) and FZD_5_ (**e**) in hRPE cells and receptors linked to Wnt signaling (**f**) in ARPE-19 induce a decrease of UOS-triggered Wnt5a transcription. Standardization was performed using β-actin and GAPDH. (**d**) Wnt5a enhanced cell death triggered by H_2_O_2_. Apoptosis was measured using Hoechst staining and ImageJ using parameters shown in (**a**). (**g**) FZD_5_ was measured in hpRPE cells undergoing UOS treated with 100 nM NPD1 or 100 ng/ml Box5 in the presence or absence of 50 ng/ml Wnt5a. The bands were standardized by total protein stain (see Materials and Methods for description). The bars represent the average of triplicates (Supplementary Fig. S2). (**h**) Deglycosylation of Wnt5a secreted by hpRPE cells. The secreted Wnt5a protein was concentrated from the medium by Chloroform/Methanol precipitation. The pellet was resuspended and digested with N and O-glycosylases (Degly). In parallel, non-digested samples (Gly) were run. Western blots were replicated using two different antibodies and a positive control (Supplementary Fig. S3). (**i-j**) Content of Wnt5a in a medium of human RPE cells in the presence of 1600 µM H_2_O_2_ and 10 ng/ml TNFα. (**i**) Exosome enrichment protocol using ultracentrifugation. (**j**) Content of Wnt5a in the different fractions of the medium. The bars represent the mean of three measurements and the standard error of the mean. *p < 0.05

**Fig. 2:**
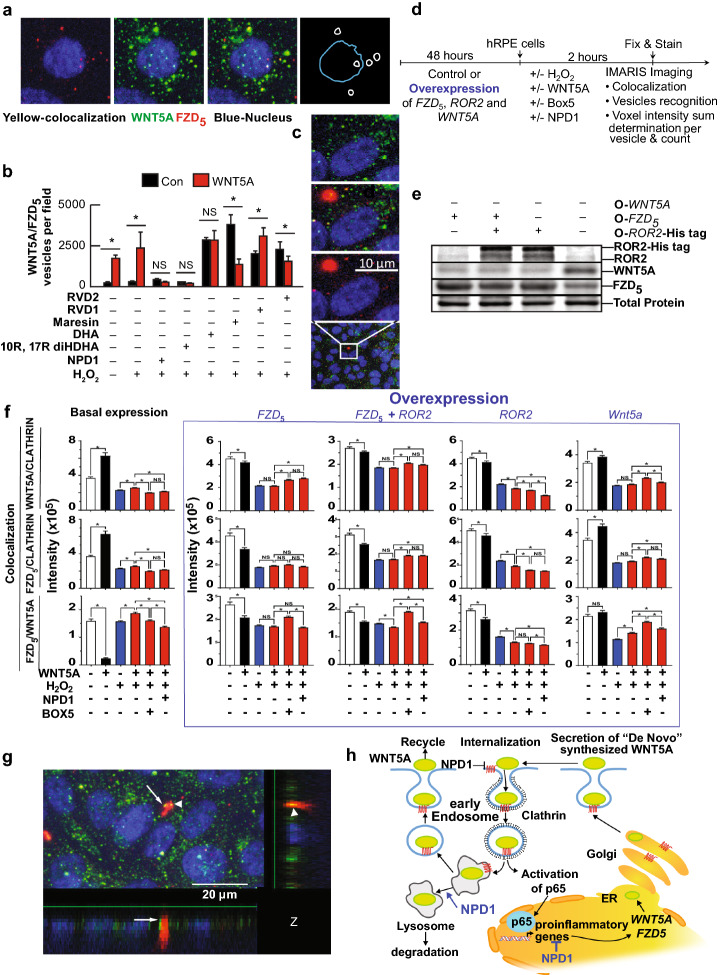
Wnt5a and FZD_5_ are internalized via CME. (**a**) Blow-up of a single cell showing vesicles positives to Wnt5a (green), FZD_5_ (red), or both (yellow). The fourth panel shows a nucleus drawing (blue) and the position of vesicles in which FZD_5_ colocalizes with Wnt5a. (**b**) Quantification of colocalized spots in RPE cells undergoing UOS ± lipids in Fig. 1b. Pearson colocalization coefficient was plotted in fig. S8D. Bars: mean of 3 measurements and standard error of the mean. * p < 0.05. (**c**) Blow-up of a cell showing large cluster of Wnt5a signal (red) present most frequently in certain treatments (Supplementary Fig. S8c). (**d**) Timeline of experiment depicted in **e** and **f**. (**e**) Western blot analysis of ROR2, FZD_5_, and Wnt5a in hpRPE cells overexpressing ROR2-His tag ORF, FZD_5_ ORF, ROR2-His tag and FZD_5_ ORFs together and Wnt5a. Duplicates of whole membranes are shown in Supplementary Fig. S4. (**f**) IMARIS analysis of the vesicles observed by immunocytochemistry targeting Wnt5a, FZD_5_, and Clathrin. The sum of the intensity for the colocalization of the signal observed for Wnt5a/Clathrin, FZD_5_/Clathrin, and FZD_5_/Wnt5a was measured with the Spots module using batch processing. Each spot corresponds to one individual or clusters of vesicles observed in hpRPE cells under UOS in the presence or absence of NPD1or Box5 and Wnt5a in the cells expressing the ORFs (open reading frame) tested by WB in (**d**). Five fields per well were averaged and computed to obtain the bars plotted. Two ways ANOVA and Tukey's HSD was applied to determine the pairwise comparison significance. The number of Spots plots for each histogram is depicted in Supplementary Fig. S5. (**g**) Vesicle-like signal in the Z axes of the Z-stack. Whole arrow shows a fusion between an FZD_5_ and Wnt5a positive to a large Wnt5a-positive cluster. Arrowhead shows an already fused colocalized cluster. (**h**) Model of internalization and recycling of Wnt5a and FZD_5_ to activate NFkB/p65. The bars represent the mean of three measurements and the standard error of the mean. *p < 0.05

**Fig. 3:**
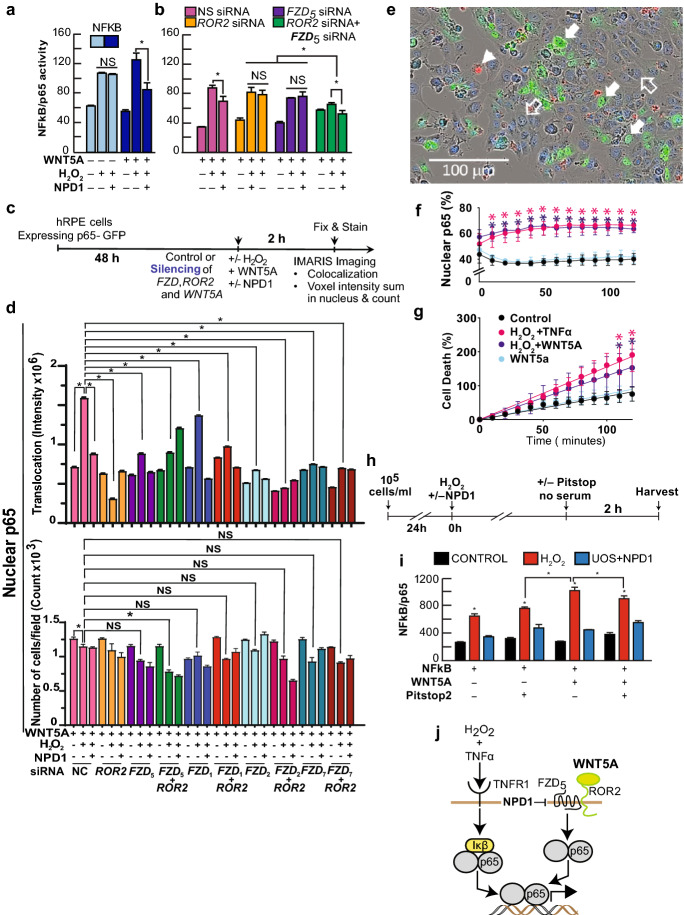
Wnt5a triggers the activation of p65 in human RPE cells undergoing UOS. (**a, b**) Luciferase assay using NFkB/p65 binding site construct (Supplementary Fig. S6) to determine its activation in the presence or absence of Wnt5a in hpRPE cells undergoing UOS plus/minus NPD1. The standardization was made co-expressing constitutively GFP. (**b**) hRPE cells were co-transfected with the NFkB/luciferase plasmid and siRNA targeting FZD_5_ and ROR2 separately and together or control non-specific siRNA. UOS was induced ± 100 nM NPD1 and Wnt5a. The yield of expression for FZD_5_ and ROR2 are presented in Supplementary Fig. S9. Bars show the mean of three measurements and the standard error of the mean. *p < 0.05. (**c, d**) Specificity of Wnt5a/FZD receptor activity on the activation of p65. (**c**) Timeline showing experimental design. (**d**) Silencing of FZD_1_, FZD_2_, FZD_5_, and FZD_7_ alone or together with ROR2 (Supplementary Fig. S10) in hpRPE cells expressing p65-GFP were treated with 50 ng/ml Wnt5a, in the presence or absence of H_2_O_2_ to induce uncompensated oxidative stress and NPD1 for two hours. Nuclear p65 was assessed by analyzing confocal Z-stack images with Imaris 9.8 to determine nuclear translocation (colocalization of GFP and Hoechst staining), Intensity (upper histogram), and number of cells (lower histogram). (**e–g**) In vivo monitoring of p65 nuclear translocation in resting hRPE cells or undergoing UOS cells and exposed to Wnt5a. hRPE cells expressing human p65fused to GFP incubated for 120 min in an Incucyte SX5 Life-cells Analysis instrument and registered every 10 min. TNFα was used as a positive control. (**e**) Cell death using Propidium Iodide (PI) (orange), total cell staining DRAQ5 (blue), and p65-GFP (green). Solid white arrows = Nuclear GFP (computed as DRAQ5/p65-GFP overlapped signal); solid white arrowheads = PI dying cells, open white arrows = normal cells. (**f**) Upper panel shows the percentage of cells depicting nuclear p65, (**g**) lower panel shows the percentage of dead cells that became permeable to PI staining. Nine fields per well for a total of 4 wells per condition were computed (**f, g**). (**d, f, and g**) 2-way ANOVA and Tukey HSD multiple comparisons were applied to obtained pairwise significance. *p < 0.05. (**h, i**) Pitstop2 halts activation of NFkB. (**h**) Experimental design. (**i**) Luciferase Reporter assay of three NFkB/p65 binding sites in tandem driving the expression of luciferase ORF; the construct was depicted in Supplementary Fig. S6. NPD1 = 100 nM and 1600 µM H_2_O_2_. (**j**) Model of alternative pathways of activation of p65 and the action of NPD1

**Fig. 4:**
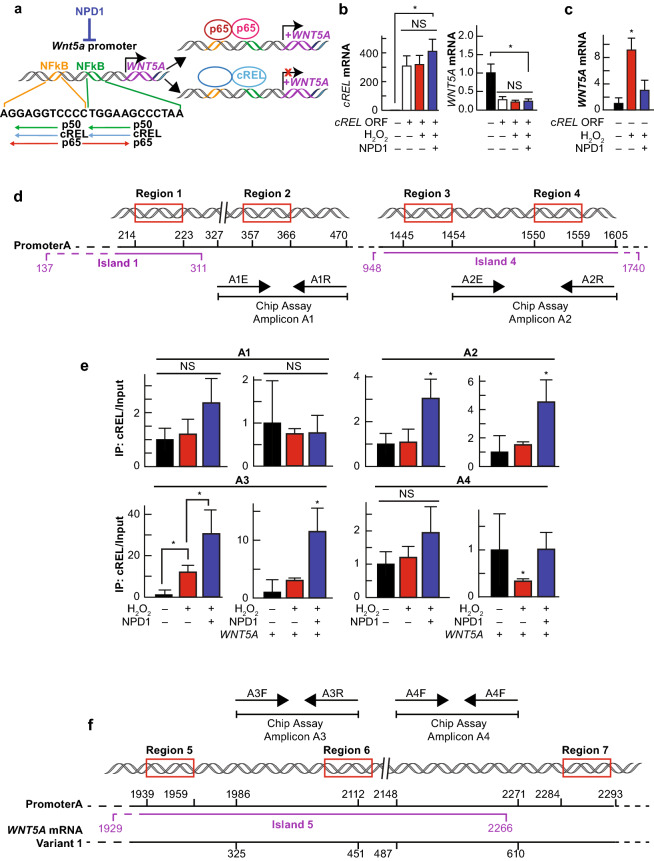
NPD1-dependent cRel binding to promoter A decreases Wnt5a expression. (**a**) Hypothesis of the role of cREL in the modulation of the expression of Wnt5a. (**b, c**) Quantification of cRel (**b**) and Wnt5a (**C**) mRNA by the means of SYBR green-based real-time PCR in hpRPE cells undergoing UOS, ± NPD1. (**B**) Cells transfected with cREL ORF were exposed to UOS in the presence or absence of NPD1 for 4 h. cREL (left) and Wnt5a (right) mRNA was quantified by real-time PCR. (**c**) Wnt5a mRNA quantification of non-transfected cells (Control for **b**). (**d, f**) Possible regulation of NFkB sites by cRel: in silica analysis of Wnt5a promoter (Katula et al. 2012) showing that the two binding sites for NFkB have high affinity for p65, p50, and cRel. The cartoon shows the possible direction in which transcription factors act. NFkB site prediction is in Supplementary Table S1. Other possible NFkB binding sites were detected by TRED. Region 2 corresponds to upstream NFkB binding site, and Region 6 to the downstream binding site depicted in** d** and **f**. Regions 1, 3, 4, 5, and 7 showed up in the general TRED search with high score (Supplementary Table S1) for the three NFkB. Four amplicons were designed close to or sitting on these regions to assess each site. CpG islands that encompass the putative binding sites are depicted in purple (Supplementary Table S2). (**e**) SYBR green-based real-time PCR using as template the proteinase digested genomic DNA fragments resulting from micrococcal DNAs digestion and cRel pull-down. 1600 µM H_2_O_2_ plus 10 ng/ml TNFα. NPD1 = 100 nM and Wnt5a 50 ng/ml unless stated otherwise. The bars represent mean of three measurements and standard error of the mean. *p < 0.05

**Fig. 5:**
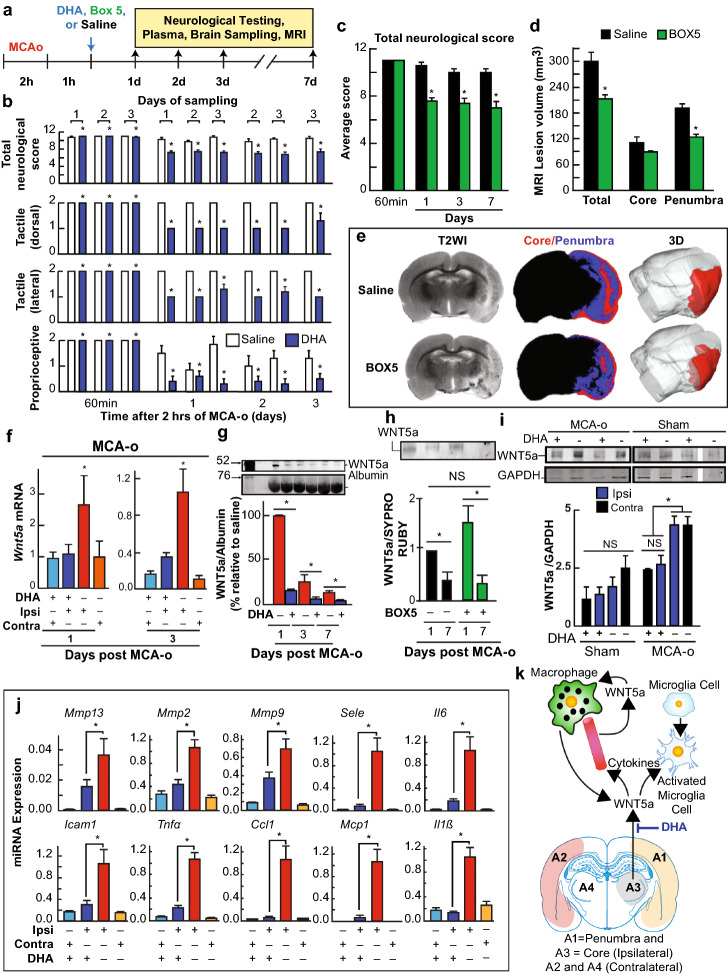
Neuroprotection by DHA prevents Wnt5a overexpression and secretion in response to brain ischemia–reperfusion and by Box5 that blocks its action. (**a**) DHA, Box5, or saline were administered at 1 h after 2 h of MCAo, and rats were sacrificed on days 1, 2, 3, or 7. (**b**) Neurological recovery. Total score (normal score = 0, maximal deficit = 12), tactile placing (dorsal, lateral, proprioceptive reactions; normal score = 0, maximal deficit = 2). DHA or saline was administered at 1 h after 2 h of MCAo, and rats were sampled on days 1, 2, 3, or 7. Values are mean ± SD; n = 4 rats/ group. *Significantly different from saline group (p < 0.05, repeated measures ANOVA followed by Bonferroni tests). (**c-e**) 400 µg Box5 IV administration, 1 h after MCAo (**c**) Total neurological score at days 1, 3, and 7; (**d**) MRI quantification at day 7 of lesion volume depicting total, core and penumbra and (**e**) representative coronal sections showing T2-weighted image (T2WI), the defined core and penumbra region (red and blue, respectively) in the second column; and a 3D reconstruction of the lesion. (**f**) Wnt5a mRNA assessment by SYBR-green real-time PCR in rat cortex Ipsilateral (Ipsi) or contralateral (Contra) of MCAo, treated with saline (vehicle) or DHA. (**g, h**) Wnt5a protein in plasma 2 h after MCAo with DHA (N = 4); (**g**) or Box5 (N = 4); (**h**) at 1, 3 and 7 days post-surgery. (**i**) Representative bands are depicted above the plot. (**j**) Wnt5a and NFkB linked gene expression in MCAo. MCAo and saline (vehicle) and DHA for 3 days (N = 3); each reaction was run in triplicate. mRNA measured using SYBR green RT-PCR. Color-coded region to test gene expression is depicted. Bars represent t mean of 3 measurements and standard error of the mean. *p < 0.05. (**k**) Wnt5a inflammatory signaling after increased abundance due to ischemia/reperfusion

**Fig. 6:**
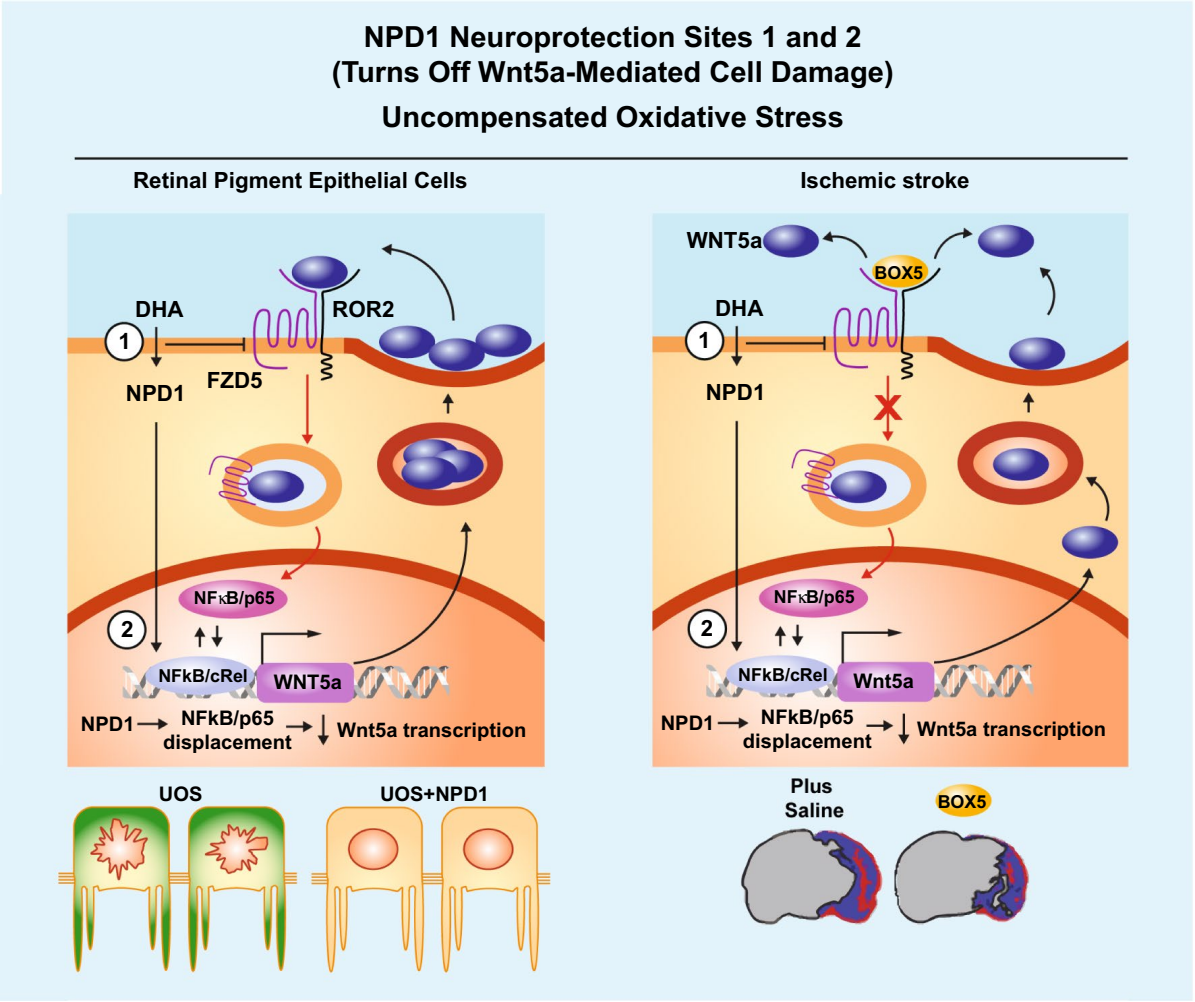
NPD1 Neuroprotection sites model. In retina pigment epithelial cells (left panel), the evidence gathered in the present manuscript points to a Wnt5a-positive feedback loop consisting of the initiation of the signaling; when Wnt5a interacts with FZD5 and ROR2, it is internalized via clathrin-mediated endocytosis, and the process elicits the activation of NFkB/p65 in cells undergoing oxidative stress conditions. NPD1 interferes with this amplification of the inflammatory signaling at two sites: 1) by reducing the availability of FZD5 (Fig. 1e, g) and thus the internalization of Wnt5a and downstream events, and 2) by increasing the availability of cREL that competes with p65 in the dimeric NFkB and decrease the transcription of Wnt5a (Fig. 4). In ischemic stroke (right panel), the reperfusion after the ischemic episode induces an oxygen-rich environment, similar to uncompensated oxidative stress status. Here, the addition of box5, a hexapeptide that competes for the binding of Wnt5a to the receptor, demonstrated that wnt5a inflammatory loop occurs under these conditions, and it is responsible for at least part of the damage observed in ischemic stroke. DHA, known to be converted into NPD1 and for activating cREL in rat brain (Calandria et al. 2015), reduced the initial increase of Wnt5a messenger in brain tissue (Fig. 5f, i) and circulated Wnt5a protein (Fig. 5g). Treatment with DHA also induced a steep decrease in proinflammatory cytokines, and SASP-related expression downstream of the Wnt5a signaling loop was in agreement with the model established in RPE cells

In Fig. 5, the caption of part i should read as “Representative bands are depicted above the plot.”

In addition, author Khanh V. Do’s present address Faculty of Medicine, PHENIKAA University, and PHENIKAA Research and Technology Institute (PRATI), A&A Green Phoenix Group JSC, Hanoi, Vietnam is included.

The original article has been corrected.

